# Biomechanical gait analysis and rehabilitation in a traumatic hallux deficit patient: a case report

**DOI:** 10.1186/s13256-024-04444-z

**Published:** 2024-03-15

**Authors:** Naoki Doi, Todd Pataky, Hiroshige Tateuchi, Momoko Nagai-Tanima, Tomoki Aoyama

**Affiliations:** Department of Physical Therapy, Human Health Sciences, Graduate School of Medicine, Kyoto University, 53, Kawahara-Cho, Shogoin, Sakyo-Ku, Kyoto, 606-8507 Japan

**Keywords:** Traumatic hallux deficit, Rehabilitation, Gait analysis, Balance training, Biomechanics

## Abstract

**Background:**

The hallux plays a crucial role in maintaining standing balance and facilitating forward and backward movements during gait.

**Case presentation:**

A 21-year-old Japanese patient, suffering from a traumatic hallux deficit with only a portion of the basal phalanx intact, underwent rehabilitation treatment. The thenar area exhibited instability, leading to impaired balance and walking difficulties. Biomechanical assessment revealed the need for a rehabilitation strategy for the foot, as well as the knee, hip, and trunk. A rehabilitation protocol was designed to enhance medial foot loading during walking and standing, including balance and trunk strength training. After a 12-week rehabilitation period, the patient’s gait showed significant improvement. Specifically, the load response and single-support phases of the gait cycle on the affected side increased from 46.9% to 49.3%, while the pre-swing phase decreased from 14.6% to 11.6%. The vertical component of the ground reaction force rose from 599.8 to 647.5 N. The enhanced stability from balance training and increased muscle strength contributed to the patient’s improved walking and balance.

**Conclusion:**

A patient with a traumatic hallux deficit underwent conservative treatment through strategic rehabilitation according to biomechanical assessment. This case report underscores the value of biomechanical gait analysis in the conservative management of similar conditions.

## Background

Toes play a vital role in maintaining postural stability while standing and walking. Notably, the hallux is instrumental in managing the body’s center of gravity in the anterior–posterior direction [1]. Reconstruction uses two to four toes [2], and thumb reconstruction uses the second toe [3]. Case reports for hallux amputation have been documented, but reports about rehabilitation are scant [4]. This study aims to present a case report of conservative rehabilitation for a traumatic hallux deficit.

## Case presentation

**Fig. 1:**
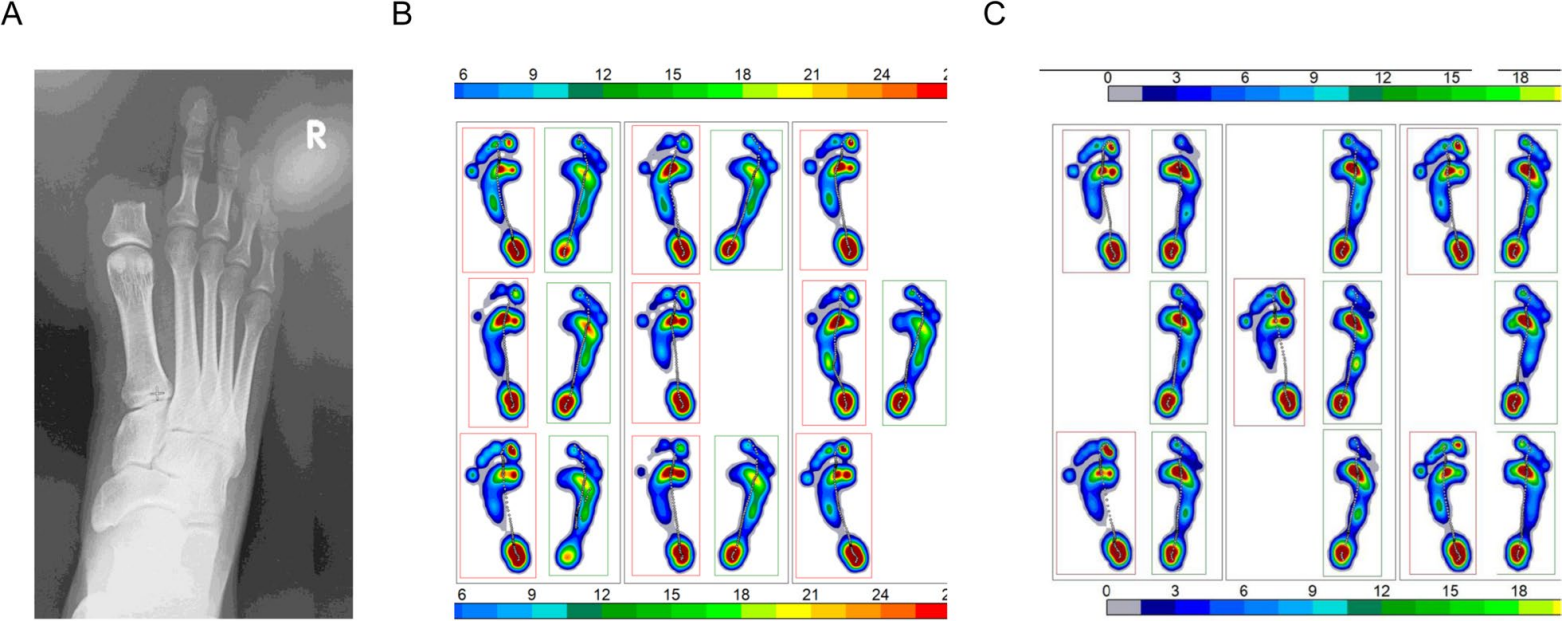
Foot pressure distribution during gait before and after rehabilitation. **A** Radiograph captured at the time of injury. **B** Prior to rehabilitation, foot pressure distribution was analyzed during walking, revealing diminished pressure in the thenar region and the medial aspect of the right foot owing to the hallux deficit. **C** Post-rehabilitation foot pressure assessment during walking indicated increased pressure in the thenar region and the medial aspect of the right foot, enhancing load distribution during movement

A 21-year-old male Japanese student, an active member of an equestrian club, sustained a right toe injury during an accident in a club-related activity. The injury resulted in the severance of his right toe above the interphalangeal joint (Fig. 1A). Following initial wound treatment, he was directed to the rehabilitation department 2 weeks post-injury. He had no remarkable medical or family history. The patient agreed to provide the case report and consented to its publication, including any accompanying images. The ethics committee of our institution waived the need for ethical review, as the patient provided written consent for the case report.

**Fig. 2:**
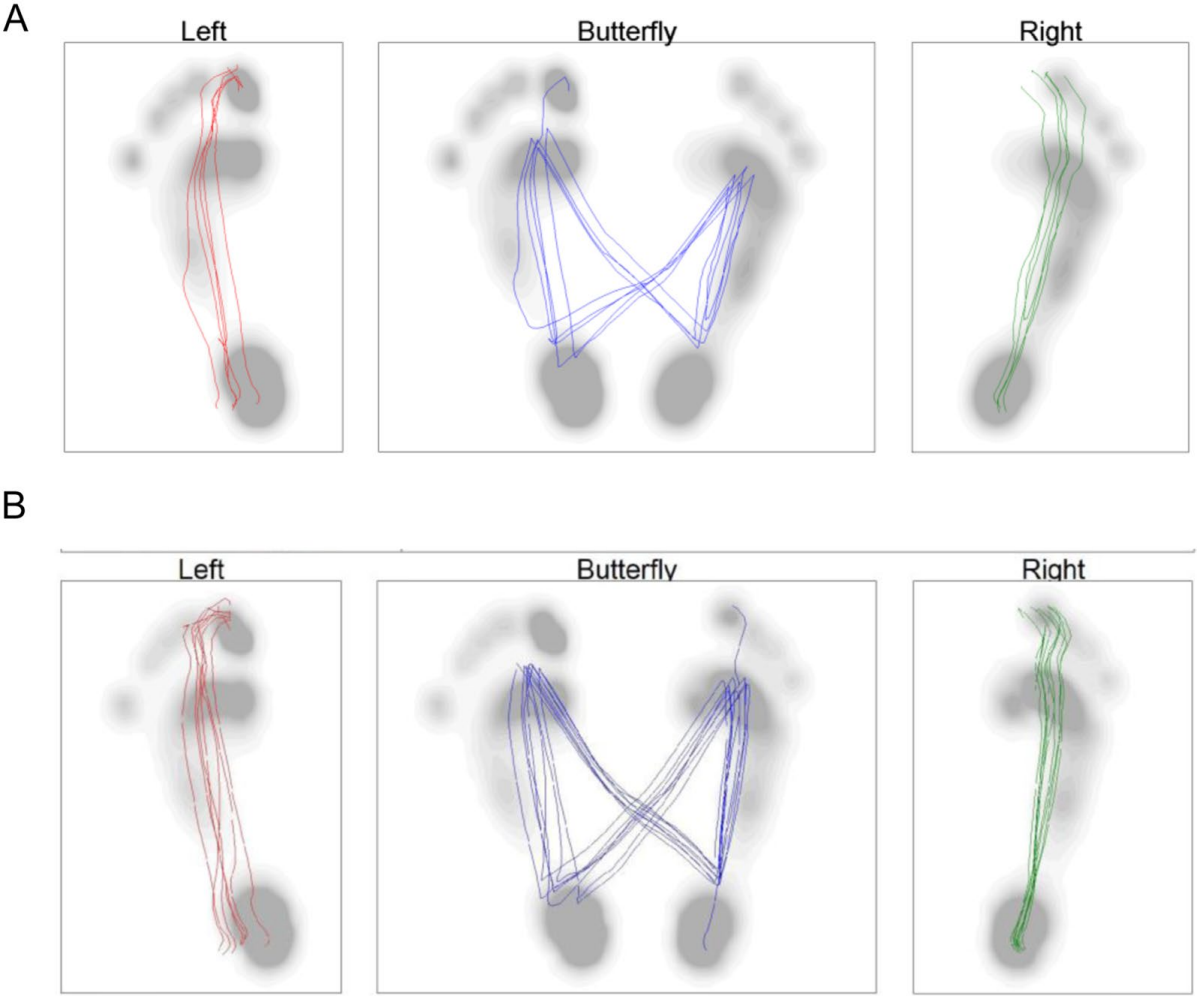
Center of gravity shifts during gait before and after rehabilitation. **A** Pre-rehabilitation analysis of gravitational force depicted shifts in the center of gravity during the terminal stance phase on the right foot, deviating toward the second toe. **B** Post-rehabilitation gravitational force analysis showed stabilization in the center of gravity’s path. The line traces the path of the center of gravity throughout the walking cycle

**Fig. 3:**
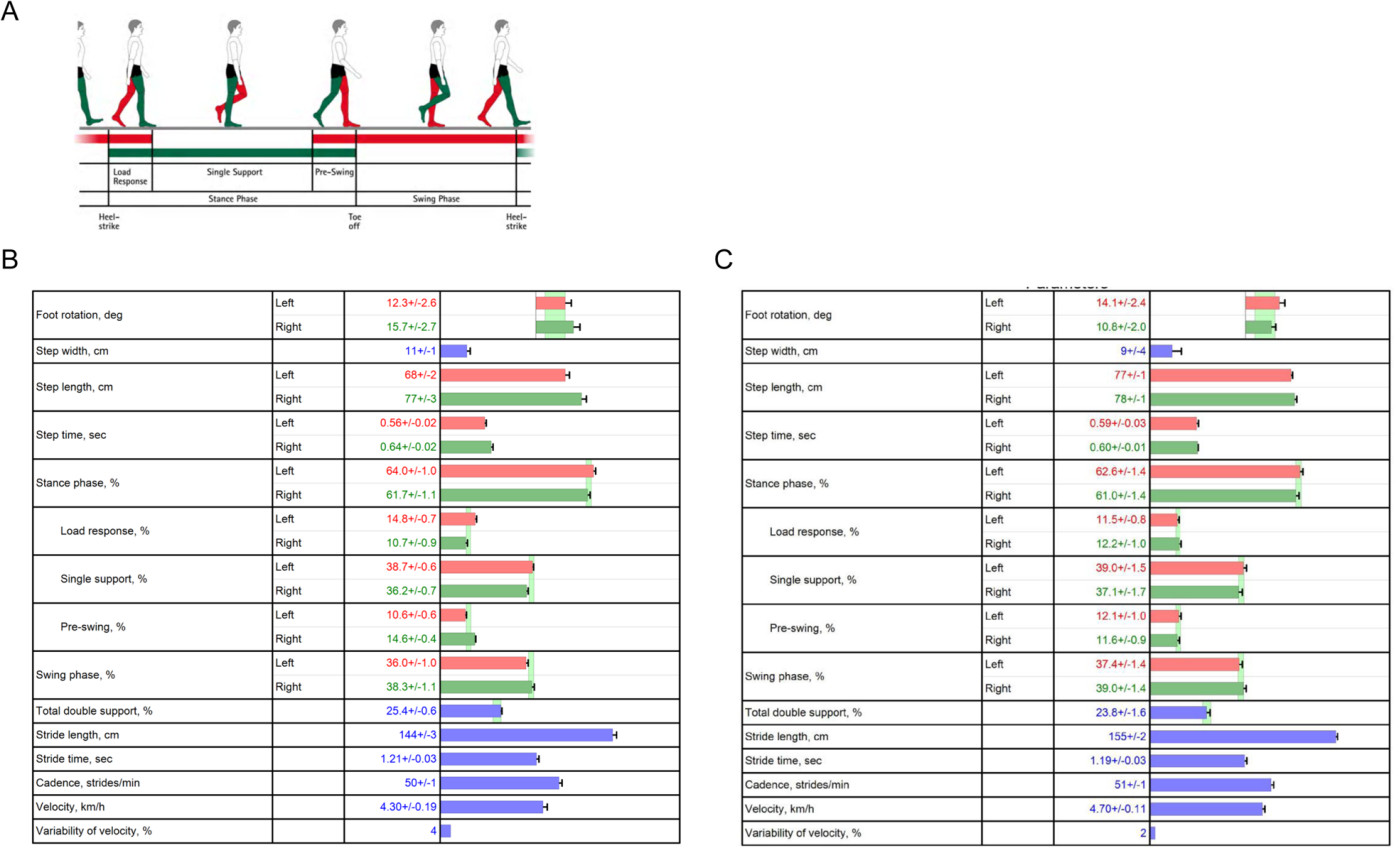
Gait cycle parameters before and after rehabilitation. **A** Gait cycle parameters. **B** Gait cycle parameters before rehabilitation demonstrated a reduction in the instantaneous phase duration of the right foot. **C** Gait cycle parameters following rehabilitation showed a decreased load response phase. The duration of the single-support phase of the right foot increased, and the asymmetry between the left and right feet diminished. The graph represents the proportion of time allocated to each phase within the gait cycle

Initial assessments indicated pain localized to the injury site, primarily during walking. One month post-injury, the patient also reported pain in the right knee joint while engaging in riding or walking. The pain in the area of the hallux deficit hindered his ability to apply pressure on the thenars. Diagnostic tests, including walking cycle analysis, temporospatial gait parameters, foot pressure distribution, ground reaction force measurements, toe grip force, and one-leg standing tests, were conducted. The foot pressure during a comfortable walk were recorded (Fig. 1B, C) using a Zebris plantar pressure platform (FDM; GmbH, Munich, Germany; number of sensors: 11,264; sampling rate: 100 Hz; sensor area: 149 cm × 54.2 cm) [5–7]. The center of pressure (COP) during a comfortable walking pace was captured using force plates (Kistler Japan Co., Ltd., Tokyo, Japan) at a sampling rate of 1000 Hz with a low-pass filter set at 20 Hz [5] (Fig. 2). A diminished foot pressure was observed not just in the hallux but also in the metatarsal head of toes two to five on the injured side (Fig. 1B). The left-foot COP shifted toward the left toe in the late stance phase, while the right-foot COP transitioned toward the second toe, exhibiting greater spatial variability (Fig. 2A). Assessing the walking cycle using a Zebris plantar pressure platform (Fig. 3), the load response and single-support durations were reduced, while the pre-swing phase was extended (Fig. 3B).

Vertical ground reaction force during the second peak of walking was diminished on the right side (right 599.8 N, left 660.3 N). The patient was unable to maintain a one-leg stand for 10 seconds on the right foot. Pain levels for the toe and knee were rated 5 out of 10 on a numerical rating scale (NRS).

**Fig. 4:**
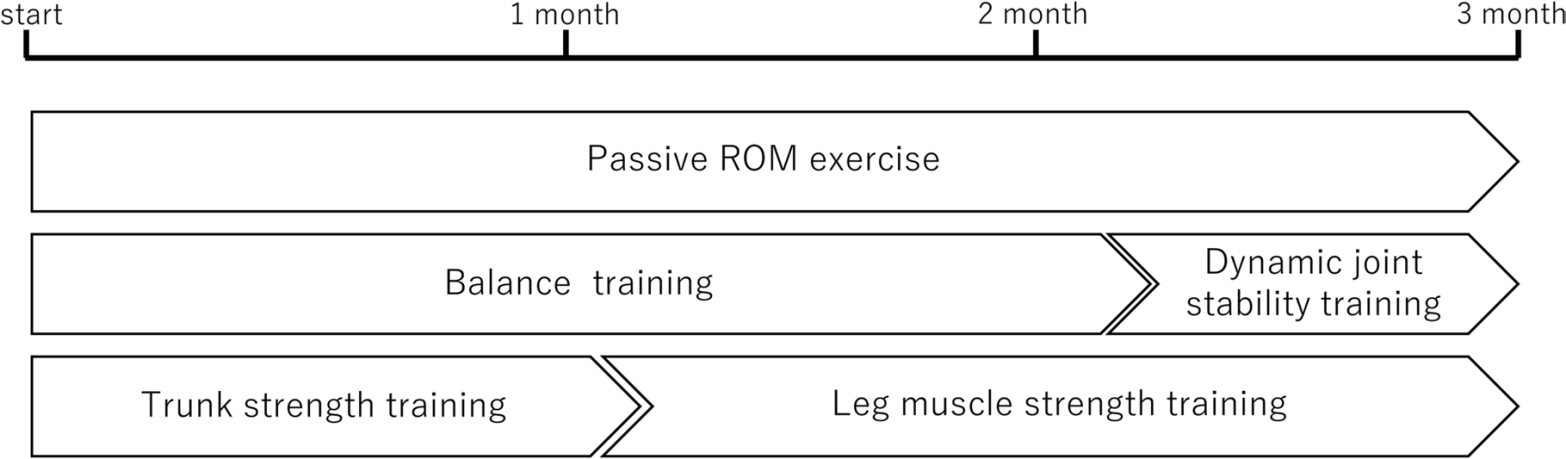
Rehabilitation protocol

On the basis of these findings, a rehabilitation protocol was designed to enhance medial foot loading and improve foot pressure during walking and standing (Fig. 4). The regimen included balance and trunk strength training. Starting from the fifth week, the focus shifted to lower limb strength training. From the ninth week, dynamic joint stability exercises were introduced to enhance neuromuscular coordination for movement stabilization [8]. After 3 months, foot pressure and COP movement during walking improved, as did pressure distribution across toes two to five and the metatarsal head, with negligible discrepancies between the left and right sides in both the vertical and anterior–posterior directions (Fig. 1C). The pressure was consistently distributed from the second toe of the right foot (Fig. 2B). Notable enhancements were also observed in the pre-leg phase (Fig. 3C). The second peak of the vertical component of the ground reaction force increased (right 647.5 N, left 639.5 N). One-leg standing on the right foot became stable for 10 seconds, even on a balance mat. NRS scores for the toe and knee pain reduced to 0, indicating a return to pre-injury levels of activity.

## Discussion

The hallux serves as a crucial anchorage point for the abductor hallucis muscle, which upholds the medial longitudinal arch [9]. This arch functions as a stabilizing structure during single-leg standing and mitigates excessive pronation during the walking stance phase. Initially, the patient refrained from putting weight on the forefoot owing to instability concerns and toe pain, as shown in Fig. 1B. This avoidance likely led to an abnormal gait and medial knee pain. Post-treatment, the improved ability to evenly distribute weight on the inner foot while walking or standing on one leg might have lessened the knee joint inversion caused by ankle supination [10], thereby alleviating knee discomfort.

The usual rehabilitation for a patient with toe deficit mainly involves a foot approach. However, in the present case, knee, hip, and trunk training were applied in addition to the foot. Lumbar lordosis and thoracic kyphosis characterize the posterior COP during standing. Previous research indicated that, during the stance phase of walking with sway-back posture, the hip flexion moment is prolonged. Additionally, activity in the tensor fasciae latae muscle is more pronounced than in the gluteus medius [11]. The presence of the Trendelenburg sign, observed when the tensor fasciae latae muscle is more active than the gluteus medius [12], is believed to contribute to COP instability while walking. In our approach, we focused on strengthening the patient’s trunk muscles and enhancing the flexibility of the lower limbs to promote dorsiflexion of the ankle joint and hip mobility, as well as to fortify spinal control through robust trunk musculature. Moreover, an increase in muscle strength might contribute to improved anterior propulsion during walking.

While there are studies on reconstructive surgery for traumatic hallux deficits [2, 3], reports on rehabilitation, particularly conservative management, are scarce. This case report discusses the conservative treatment of a traumatic hallux deficit resulting from an injury, aimed at enhancing posture and gait. The assessment and rehabilitation techniques applied in this study could offer valuable perspectives for the future conservative management of traumatic hallux deficits.

## Conclusion

A patient with a traumatic hallux deficit underwent conservative treatment through rehabilitation. The rehabilitation protocol was strategically designed to enhance left–right load balance and COP dynamics in the anterior–posterior direction, leading to an improved load balance and successful reacquisition of gait. The application of biomechanical gait analysis has proven to be beneficial in managing such cases.

## Data Availability

Not applicable.

## Abbreviations

COP: Center of pressure
NRS: Numerical rating scale
